# Real-Time Road Intersection Detection in Sparse Point Cloud Based on Augmented Viewpoints Beam Model

**DOI:** 10.3390/s23218854

**Published:** 2023-10-31

**Authors:** Di Hu, Kai Zhang, Xia Yuan, Jiachen Xu, Yipan Zhong, Chunxia Zhao

**Affiliations:** 1 School of Computer Science and Engineering, Nanjing University of Science and Technology, Nanjing 210094, China; hudi@njust.edu.cn (D.H.); kaiopen@njust.edu.cn (K.Z.); bbxujiachen@njust.edu.cn (J.X.); zhaochx@njust.edu.cn (C.Z.); 2 Research Institute of Intelligence, Southwest Research Institute of Information Control, Chengdu 611700, China; zyp_cse@njust.edu.cn

**Keywords:** intersection detection, 3D point cloud, augmented viewpoints, bird’s eye view

## Abstract

Road intersection is a kind of important navigation landmark, while existing detection methods exhibit clear limitations in terms of their robustness and efficiency. A real-time algorithm for road intersection detection and location in large-scale sparse point clouds is proposed in this paper. Different from traditional approaches, our method establishes the augmented viewpoints beam model to perceive the road bifurcation structure. Explicitly, the spatial features from point clouds are jointly extracted in various viewpoints in front of the robot. In addition, the evaluation metrics are designed to self-assess the quality of detection results, enabling our method to optimize the detection process in real time. Considering the scarcity of datasets for intersection detection, we also collect and annotate a VLP-16 point cloud dataset specifically for intersections, called NCP-Intersection. Quantitative and qualitative experiments demonstrate that the proposed method performs favorably against the other parallel methods. Specifically, our method performs an average precision exceeding 90% and an average processing time of approximately 88 ms/frame.

## 1. Introduction

**Figure 1:**
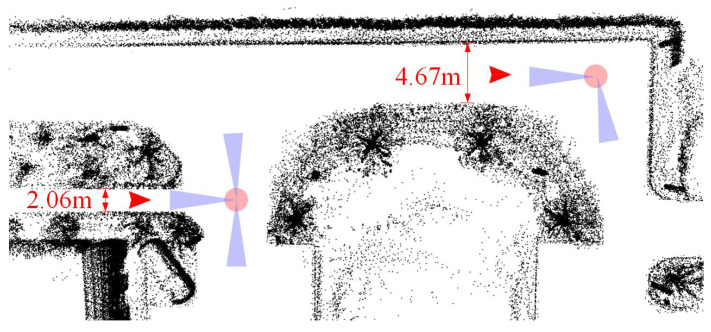
Intersection detection and location by Velodyne VLP-16. The red arrow indicates the pose of the UGV.

An unmanned ground vehicle (UGV) should find out when and where to turn while navigating in a city. As a result, the intersection becomes a kind of crucial landmark. Intersection detection under normal traffic conditions is a challenging perception task due to the variability in scenarios [1]. The current intersection detection methods can be categorized into two main classes: learning-based [2,3,4,5,6] and learning-free [7,8,9,10,11] approaches. The former methods typically combine various types of data sources, including images, point clouds, GPS, and trajectories. However, they tend to struggle in real-life situations when only a single data source is available. On the other hand, the latter approaches primarily rely on the traditional algorithms for enhancing intersection detection based solely on point clouds. The beam model algorithm has been widely employed in road intersection detection due to its remarkable ability to extract spatial features. However, existing algorithms based on the beam model exhibit limitations in terms of robustness when confronted with intersections of varying structures. These limitations stem from the lack of postprocessing methods after obtaining beam model data and the algorithm’s inability to perform online learning. To overcome these challenges, this paper proposes a novel lidar-based method to detect and locate intersections. It excels in maintaining real time and robustness when confronted with dynamic road intersection scenarios, as presented in Figure 1.

Learning-based approaches detect the intersections by recognizing scene features, which can quickly identify the characteristics of road boundaries [12,13,14,15,16,17,18,19]. Hata [12] identified road intersections by matching the road boundary data with the pre-defined intersection model. This method is only applicable to structured roads with complete road boundaries. Furthermore, there are also video-based methods [15]. However, the actual environment is accompanied by interference, like overexposure or direct sunlight. What is more, there exist road intersection detection methods in the field of remote sensing images [16]. These methods detect road intersections through remote sensing images and are not suitable for general mobile robots. That makes these methods difficult to adapt to the environment. Generally, these methods face the lack of local features and bear high computational overhead.

In addition, there also exist intersection detection algorithms without machine learning methods. Thrun [20] firstly proposed the beam model and successors developed related methods [7,8,10,11]. Wang [11] designed a wave crest search algorithm to extract the angle characteristics of intersection bifurcation from the output of the model. Although the processing time is fast, its detection results completely depend on the current unique detection results, and the reliability is poor. Zhang [10] proposed a sliding beam model to eliminate the dependence on detection distance parameters, while the relationship between adjacent test results is not considered in this method. Wang [9] proposed a double-layer beam model to detect the boundary and shape of the road. However, the robustness of these methods is still poor, and the excellent performance is dependent on structured road conditions. Due to the inability to fully account for the influence of obstacles on detection results, these methods lack robustness in diverse road conditions.

In certain circumstances, such as caves or buildings, data sources often possess only point clouds due to environmental and equipment limitations. Under such conditions, a UGV usually cannot observe the complete intersection shape [21,22]. Even when positioned at the center of an intersection, it may be obstructed by various obstacles within the channel. Another problem lies in how the UGV can determine the quality of its intersection detection results [23,24]. The above problems bring great challenges to the intersection detection task. Aiming to address the aforementioned issues, this paper introduces a real-time intersection detection method based on lidar. It is capable of robustly detecting and locating intersections, even in sparse point cloud data.

To summarize, the main contributions of this paper are threefold:

(1) We propose an augmented viewpoints beam model and design a real-time intersection detection method based on the model. Experiments on VLP-16 and HDL-64 lidar data show that the algorithm works well in real traffic conditions.
(2) We design online evaluation metrics to evaluate the quality of the intersection detection results. It enables the UGV to self-assess the quality of the intersection detection in real time during moving.
(3) We have collected and annotated a VLP-16 point cloud dataset specifically for intersections on our UGV, called NCP-Intersection. The dataset is publicly available at https://github.com/GetsonHu/NCP-Intersection.git (accessed on 15 August 2023).

## 2. Method

**Figure 2:**
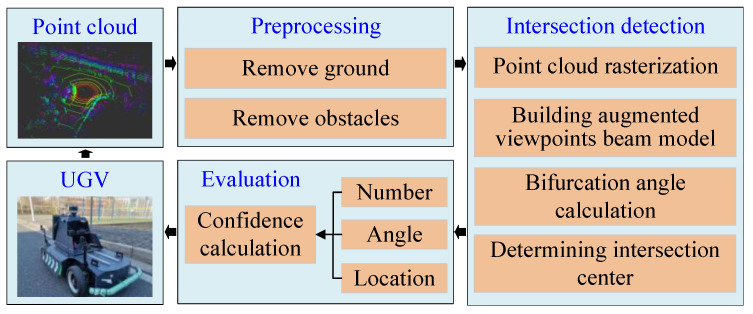
Overall structure of proposed method based on sparse point cloud. Our method consists of three main parts: (1) data preprocessing; (2) intersection detection; and (3) evaluation. In the data preprocessing phase, raw point clouds are processed to eliminate ground and obstacles. The intersection detection module utilizes an augmented viewpoints beam model to accurately perceive the road bifurcation structure. It enhances the detection effects by establishing multiple virtual viewpoints in front of UGV as the center of beam model. Finally, factors such as the number, angle, and location of detected intersections are taken into consideration to determine the level of confidence. This self-assessment capability allows our method to evaluate the quality of detection results.

The flowchart of our intersection detection method is presented in Figure 2. The proposed method takes a raw sparse point cloud as input and outputs the position of intersection as well as the bifurcation angle. It can be divided into three steps: step 1 involves preprocessing point clouds by removing the ground and obstacles; step 2 focuses on intersection detection and location; and step 3 entails an online evaluation of the calculation results.

### 2.1. Preprocessing

The purpose of preprocessing is to remove the ground and obstacles to reduce the false detection rate. For the initial data processing, we start by isolating the relevant region of interest (ROI). To remove the influence of the ground, the RANSAC method [25] is employed. However, directly fitting the entire ground plane using RANSAC proves challenging due to the irregularities and unevenness of the road surface. As a result, we utilize RANSAC to fit specific areas of the road surface, resulting in a set of fitted screens. Utilizing this set, we calculate the angle between each plane’s normal vector in the set and the *Z*-axis of the lidar. If the angle falls below a certain threshold, it indicates that the plane is part of the ground surface. After the ground detection process, we can separate the ground point clouds from the initial point clouds and remove the interference caused by the ground.

**Figure 3:**
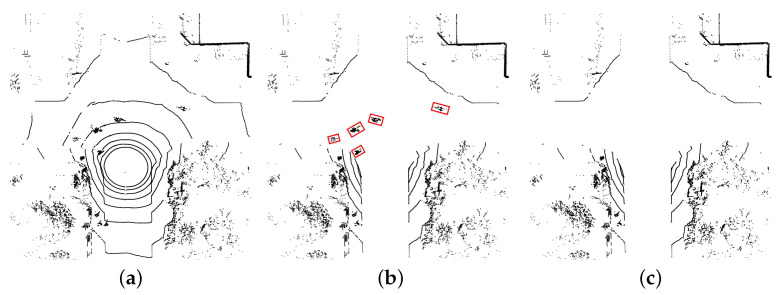
Steps of preprocessing. (**a**) is the raw sparse point cloud data. (**b**) is the point clouds after removing ground. (**c**) is the bird’s eye grid map M after removing obstacles.

Subsequently, we proceed to distinguish between the ground and non-ground regions on the two-dimensional projection map along the *Z*-axis of the lidar. Within the ground area, the above-ground point clouds often manifest as a cluster of isolated points. To address this, we employ the Breadth-First Search (BFS) algorithm to cluster these isolated points together. As a result, individual isolated points are grouped into distinct obstacles such as pedestrians, bicycles, or cars, which need to be removed. Finally, the point clouds set **P** without the ground and obstacles can be achieved. The results of the preprocessing are shown in Figure 3. We mark the obstacles with red boxes in Figure 3b.

### 2.2. Augmented Viewpoints Beam Model-Based Intersection Bifurcation Detection

Algorithm 1 shows the process of intersection bifurcation detection based on the augmented viewpoints beam model. Based on different viewpoints, we calculate *n* groups of bifurcation angles. We then screen and select the optimal bifurcation angles as the detecting result, guided by several thresholds. In this algorithm, $S_{i}\left(i=1,2,\ldots ,n\right)$ saves the bifurcation angles detected by all the beam models, and $S_{f}$ saves the final bifurcation angles of the intersection.
**Algorithm 1** Intersection bifurcation detection**Require:** point cloud set **P**
**Ensure:** bifurcation angle set $S_{f}$
1:**Initialization:** grid width $d_{w}$ = 0.2 m, the number of single beam models *n*, distance between adjacent models $d_{m}$, beam ray resolution $\gamma_{a}$, angle set $C_{t}$, calculated bifurcation angle set $S_{f}$, maximum slope threshold $T_{s}$, minimum proportion threshold $T_{g}$2:establish the empty grid map **M** based on $d_{w}$3:**for** *k* in size(**P**) **do**4:      **if** number of point clouds in the grid > 0 **then**5:            mark the grid as occupied grid6:      **else**7:            mark the grid as unoccupied grid8:      **end if**9:**end for**10:set distance *d* in front of UGV as the center of beam model11:**for** *i* in *n* **do**12:   establish single beam model based on beam center and $\gamma_{a}$13:   figure out bifurcation angle set $S_{i}$ on current beam model14:   record the beam center coordinate $x_{i}$ and $y_{i}$, $d=d+d_{m}$15:**end for**16:arrange $S_{i}$ in ascending order17:**for** every angle $\alpha_{i}\in S_{i}$ **do**18:      **if** slope difference of $\alpha_{j}$ and $\alpha_{j-1}<T_{s}$ **then**19:          add $\alpha_{j}$ to $C_{t}$20:      **else if** the quantity proportion of $C_{t}\geq T_{g}$ **then**21:          add bifurcation angle $\beta_{f}$ = average $\left(C_{t}\right)$ to $S_{f}$, $C_{t}=⊘$22:      **end if**23:**end for**


#### 2.2.1. Grid Map-Based Single-Viewpoint Beam Model

In the first step of our method, the point clouds **P** after removing the ground and obstacles need to be rasterized. Based on the grid width $d_{w}$ = 0.2 m and region of interest (ROI), the initial grid map **M** can be created. Then, the grids are filtered by checking if there are point clouds present or not, effectively dividing them into occupied and unoccupied grids. This step projects the three-dimensional point clouds onto a two-dimensional plane, preserving all the intersection features while minimizing the computational loss.

**Figure 4:**
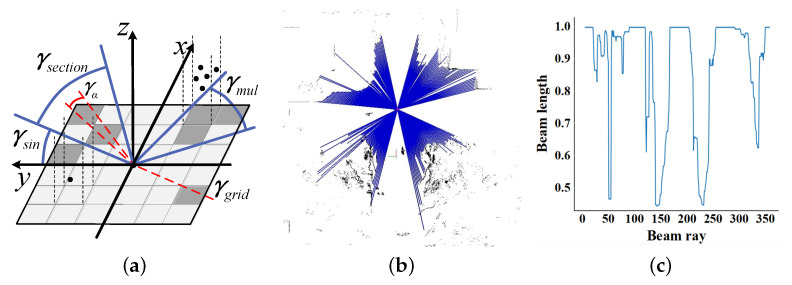
Schematic diagram of traditional beam model. (**a**) is the beam sections projected on the grid map. (**b**) is the detection effects of single beam model. (**c**) is the relationship between beam length and corresponding beam ray. There exist several groups of continuous angle regions with beam length of 1. Each group should be a reasonable candidate angle set for bifurcation.

In the beam model, the viewpoint is firstly determined as the center of the model. And the beam is defined as the connection between the viewpoint and occupied grids. As shown in Figure 4a, $\gamma_{section}$ represents the division angle section corresponding to some grid in the occupied grid map. We set $\gamma_{a}=1^{\circ }$ as the beam ray resolution, which represents the angular spacing of the adjacent beam rays. In grid map **M**, each beam ray $\gamma_{grid}$ of the grid $m_{s,t}$ is calculated as follows:

(1) $$\gamma_{grid}=arctan\frac{t-origin_{c}}{s-origin_{r}}$$

where ($origin_{r},origin_{c}$) is the position of the beam model’s origin in **M**. For all the occupied grids $m_{s,t}$ with the same beam ray, the division angle section $\gamma_{section}$ is defined as:

(2) $$\gamma_{section}=\left\{m_{s,t}=\left(s,t\right)|\left(k-1\right)<\gamma_{s,t}*\frac{180}{\pi }\leq k\right\}$$

where k∈{1,2,3,…,360}. At the same time, each beam ray in $\gamma_{section}$ corresponds to a beam length $l_{k}$, and the definition is:

(3) $$l_{k}=\min_{m_{s,t}\in Z_{k}}\sqrt{{\left(s-origin_{r}\right)}^{2}+{\left(t-origin_{c}\right)}^{2}}$$

As is shown in Figure 4a, section $\gamma_{mul}$ contains multiple occupied grids, and we use the Euclidean distance between the shortest grid and the central grid as the beam length of $\gamma_{mul}$. If the section contains only one grid like $\gamma_{sin}$, the beam distance is determined by the occupied grid.

After normalization, the beam length $l_{k}$ is quantified as a value between 0 and 1. For the division angle section without an occupied grid, $l_{k}$ is recorded as 1. It indicates that there is no occlusion from this angle, and there may exist bifurcation in this direction. The relationship between the beam length and beam ray is shown in Figure 4c. We set the threshold $L_{c}$ to screen out the qualified beam length fragment as the bifurcation angle.

#### 2.2.2. Augmented Viewpoints Beam Model

**Figure 5:**
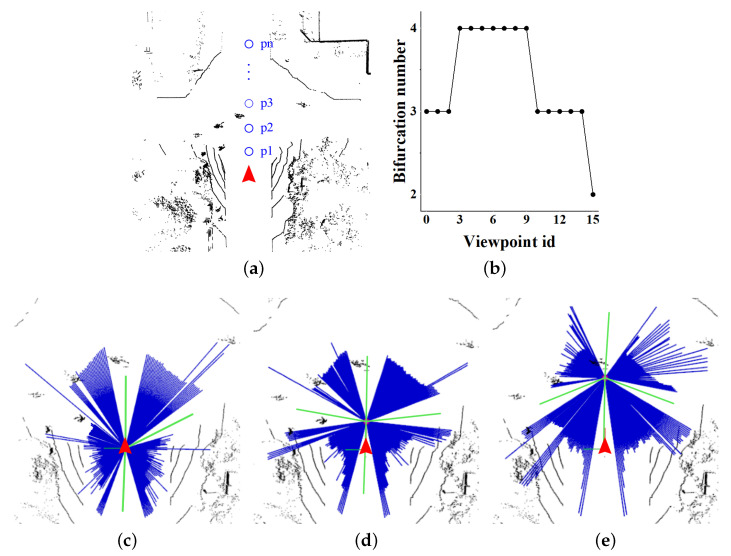
The red arrow in (**a**) represents UGV, and the blue circle indicates the virtual viewpoints in front of UGV. The relationship between bifurcation number and viewpoint id are shown as (**b**). The sketch map of the augmented viewpoints beam model in single frame of point cloud is shown as (**c**–**e**), and green lines indicate the calculated bifurcations.

With a single-viewpoint beam model, we place *n* virtual viewpoints $\left(p_{1},p_{2},\ldots ,p_{n}\right)$ in front of the UGV as is shown in Figure 5a. They are applied as the origins to establish multiple beam models in one frame of point clouds. We set two thresholds $T_{W}$ and $T_{L}$ in advance to filter bifurcations. $T_{W}$ represents the minimum angle range covered by a single angle set, and $T_{L}$ indicates the maximum angle difference between two adjacent angle sets. The interval of the viewpoints is $d_{m}$, and the position of the *i*-th viewpoint is defined as:

(4) $$\left\{\begin{array}{cc} x_{i}=origin_{r}-i*\frac{d_{m}}{d},\;i\in 1,2,\ldots ,n & \\ y_{i}=origin_{c} \end{array}\right.$$

Similar to the traditional beam model, the detection results of a single-frame point cloud generated by an augmented viewpoints beam model are shown in Figure 5. As parameter *i* increases, the distance between the corresponding beam center and the UGV becomes larger. Without reaching these beam centers, each beam model corresponds to its own bifurcation results. Repeating the above single beam model *n* times, we obtain *n* groups of intersection bifurcation angles. With the maximum slope threshold $T_{s}$ and minimum proportion threshold $T_{g}$, the final intersection angle set is filtered.

### 2.3. Determining Intersection Center

Based on the bifurcation angle sets, we can figure out the center of the intersection location. The key problem is to determine the distance between the intersection and the UGV. In the robot coordinate system, the position of the center of the intersection refers to the position of the *i*-th model in front of the UGV. Based on the intersection bifurcation angle set, the bifurcation angle of the same intersection is very close in theory. Therefore, even if there are missed or false detections, the other correct angles still have high similarity.

After the above operations, we can obtain the bifurcation angle set $S_{i}$ in each single beam model and the final intersection bifurcation angle set $S_{f}$. For every element $s_{ij}$ in $S_{i}$, we find its closest element $s_{fi}$ in $S_{f}$. Then, the degree of separation $dd_{i}$ between two bifurcation angle sets is defined as:

(5) $$dd_{i}=\frac{1}{|S_{i}|}\sum_{s_{ij}\in S_{i},s_{fi}\in S_{f}}\left|s_{fi}-s_{ij}\right|$$

where $|S_{i}|$ indicates the number of angles in $S_{i}$. It is applied to reduce the effects of possible false detection angles. Selecting the origin of the beam model with the smallest $dd_{i}$, the relative position of the intersection center can be achieved.

### 2.4. Confidence Evaluation

In this section, we design a metric to measure the quality of the detection results online. Fully considering the interfering factors of our method, the three aspects below are considered.

#### 2.4.1. The Number of Bifurcations

During the process of intersection detection, false detection will lead to the local jump in the number of intersection bifurcations. Aiming at this problem, we set the confidence $c_{1}$:

(6) $$c_{1}=m/n$$

where *m* indicates the number of beam models for calculating the correct bifurcation number, and *n* indicates the total number of beam models.

#### 2.4.2. The Angle of Bifurcations

Based on Equation (5), we achieve the smallest degree of separation $dd_{min}$. It reflects the influence of false detection on the final intersection bifurcation angle set. The higher the value, the less credible the result is. Therefore, we set the confidence $c_{2}$:

(7) $$c_{2}=1/dd_{min}$$

#### 2.4.3. Intersection Location Matching

According to the detection properties of our method, the detection results of the robot and the detection distance show a Gaussian distribution. The separation degree and detection distance of the bifurcation angle set can be approximately fitted as an opening-upward quadratic curve. The extreme point will be the best matching point of the intersection position. After calculating the detection distance of the best matching point $i_{min}$, we compare it with the relative position of the intersection $i_{r}$. As the two values approach, the detection results will be reliable. The confidence $c_{3}$ is designed as

(8) $$c_{3}=1/\left|i_{r}-i_{min}\right|$$

Considering the above three aspects as a whole, we weigh and add the above three confidence results. The final evaluation function C(R) is expressed as:

(9) $$C\left(R\right)=\lambda_{1}c_{1}+\lambda_{2}c_{2}+\lambda_{3}c_{3}$$

where $\lambda_{1}$, $\lambda_{2}$, and $\lambda_{3}$ are weighting coefficients. By evaluating the detection results, the optimal intersection detection results can be obtained when the UGV passes through the intersection. If there are obstacles such as pedestrians or bicycles ahead, the detection results with high confidence can be maintained. This will contribute to providing more reliable intersection information for subsequent navigation.

## 3. Experiments

### 3.1. Experimental Data and Environment

**Figure 6:**
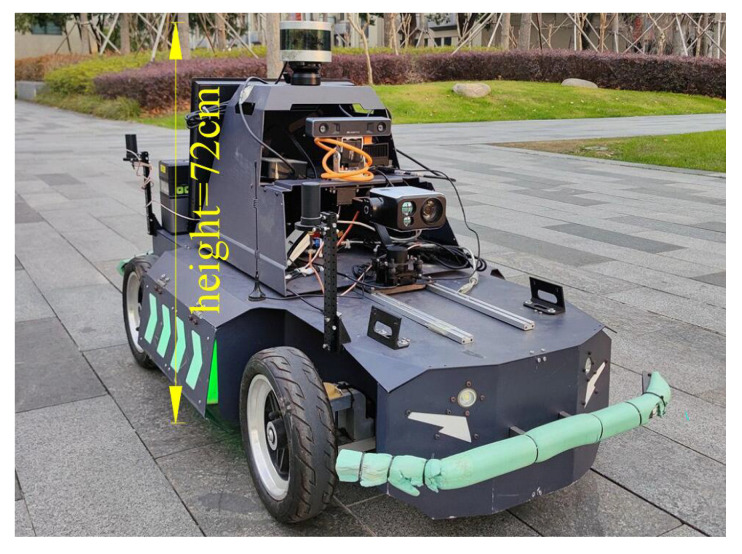
The experimental platform. The vehicle is equipped with a Velodyne VLP-16 Lidar, an inertial measuring unit, and a global navigation satellite system. The lidar is mounted on the top of the vehicle with a height of 72 cm.

The proposed method is verified on two actual scene datasets, including KITTI-raw and NCP-Intersection. In addition, the simulation experiments in Gazebo and Carla are conducted to simulate both indoor and outdoor scenes. A total of 1000 frames are evaluated in this study. The selected data in KITTI-raw contain straight, T-shaped, Y-shaped, and +-shaped intersections, with a total of 200 frames. NCP-Intersection is a dataset we collected and annotated in a campus environment with our robot, which is shown in Figure 6. Due to the impacts of the vertical scanning angle of the lidar and the road width on detection performance, we have placed restrictions on the installation height of the lidar. Because our collected data primarily pertain to campus roads with a width of approximately 6.5 m, we have determined the lidar height from the ground to be 0.72 m.

NCP-Intersection includes straight, T-shaped, +-shaped, and L-shaped intersections, with a total of 500 frames. The point cloud is collected by Velodyne VLP-16 Lidar in NCP-Intersection and is much sparser than the point cloud in KITTI-raw. A DGPS is used to obtain the ground truth location of the intersection. The robots in both Gazebo and Carla are also equipped with Velodyne VLP-16 Lidar. For each simulation environment, we select 150 frames of intersections for further analysis and evaluation. The proposed algorithms in the actual scenes are tested both on an Intel i5-8259U and 8-core ARM CPU, respectively.

### 3.2. Ablation Study of Parameters

**Figure 7:**
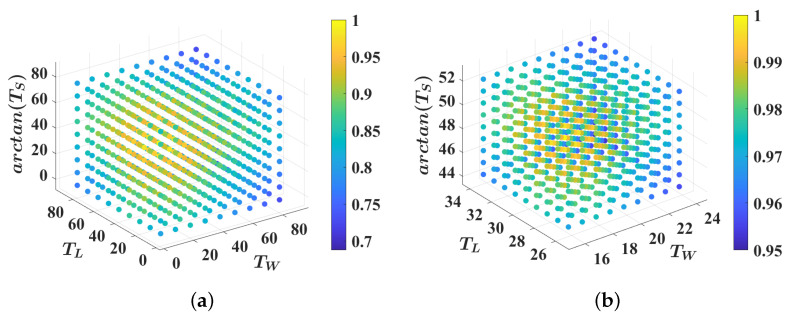
Results of grid search for three parameters $T_{W}$, $T_{L}$, and $T_{s}$. As depicted in (**a**), the optimal value of TSR is achieved when $T_{W}\in \left(15,25\right)$, $T_{L}\in \left(25,35\right)$, and $T_{s}\in \left(45,55\right)$. To determine the optimal combination, we further restrict the range of these three parameters and conduct a grid search again, as illustrated in (**b**).

The proposed method considers the parameters $T_{W}$, $T_{L}$, and $T_{s}$ as crucial factors that influence the detection outcomes. The different combinations of these parameters have a direct impact on the method’s performance. To determine the optimal combination, the True Segmentation Rate (TSR) [10] is used as the evaluation metric. Figure 7 and Table 1 present the objective evaluation values obtained from the various combinations tested on the NCP-Intersection dataset. In Figure 7, we conduct a grid search and exhaustively list all the possible values for the three parameters. As shown in Figure 7a, the detection performance is the best in the central area. Therefore, we narrow down the parameter value range, as shown in Figure 7b. Referring to Table 1, it is evident that our method achieves the best performance when $T_{W}=20$, $T_{L}=30$, and $T_{s}=tan49$. Consequently, based on this analysis, we adopt $T_{W}=20$, $T_{L}=30$, and $T_{s}=tan49$ in the framework.

### 3.3. Results and Analysis

**Figure 8:**
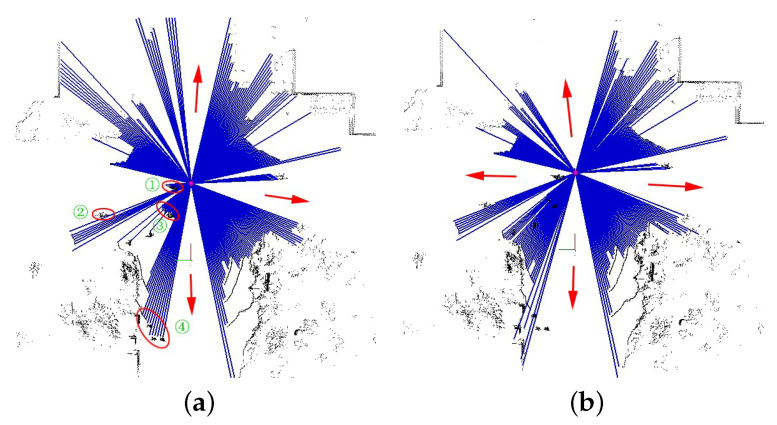
Intersection detection w/o removing obstacles. (**a**) +-shaped intersection detection without removing obstacles. (**b**) +-shaped intersection detection with removing obstacles.

Firstly, we verify the necessity of preprocessing. Figure 8 shows the importance of obstacle removal in accurately detecting the number of bifurcations. Without removing the obstacles, the beam model incorrectly detected the +-shaped intersection as being T-shaped (Figure 8a). The detection result is correct after removing the obstacles and accurately reflects the actual intersection shape (Figure 8b).

**Figure 9:**
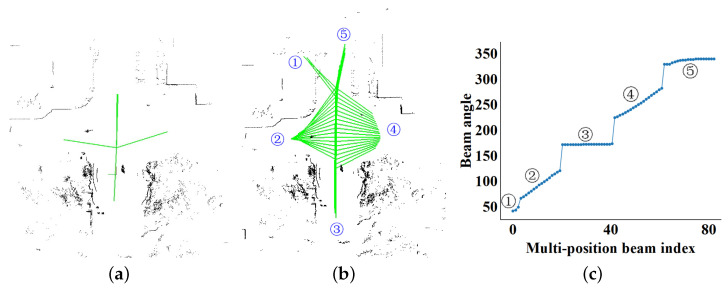
The green ray in (**a**) represents the bifurcation orientation in single-viewpoint beam model. After *n* times, we identify five orientations, which are shown in (**b**,**c**).

Figure 9 shows the statistical results of the bifurcation set detected by the augmented viewpoints beam model. Based on the angle set of the bifurcation in Figure 9c, we find that segments ③ and ⑤ with the slope close to the horizontal correspond to the front and rear bifurcations. The left and right bifurcations correspond to segments ② and ④. However, the algorithm also detects ① by mistake as the bifurcation. With filtering through $T_{s}$ and $T_{g}$, it considers ① as an obstacle interval and eliminates the possibility of it being a bifurcation.

To demonstrate the superiority of our proposed method, we conduct a comparative analysis with five parallel approaches that employ enhanced algorithms for the beam model. These approaches include methods proposed by Zhu [8], Chen [7], Zhang [9], Zhang [10], and Wang [11]. The approaches proposed by Zhu [8] and Chen [7] employ a single beam model, where a single viewpoint is established to construct the beam model and extract intersection features. Zhang [9] introduces a double-layer beam model to recognize the intersection shape and classify the road type. The method proposed by Zhang [10] utilizes a sliding beam method for road segmentation. Lastly, the approach proposed by Wang [11] extracts edge feature points from the point cloud data using height, flatness, and horizontal error as criteria and then utilizes the beam model to perform intersection detection.

**Figure 10:**
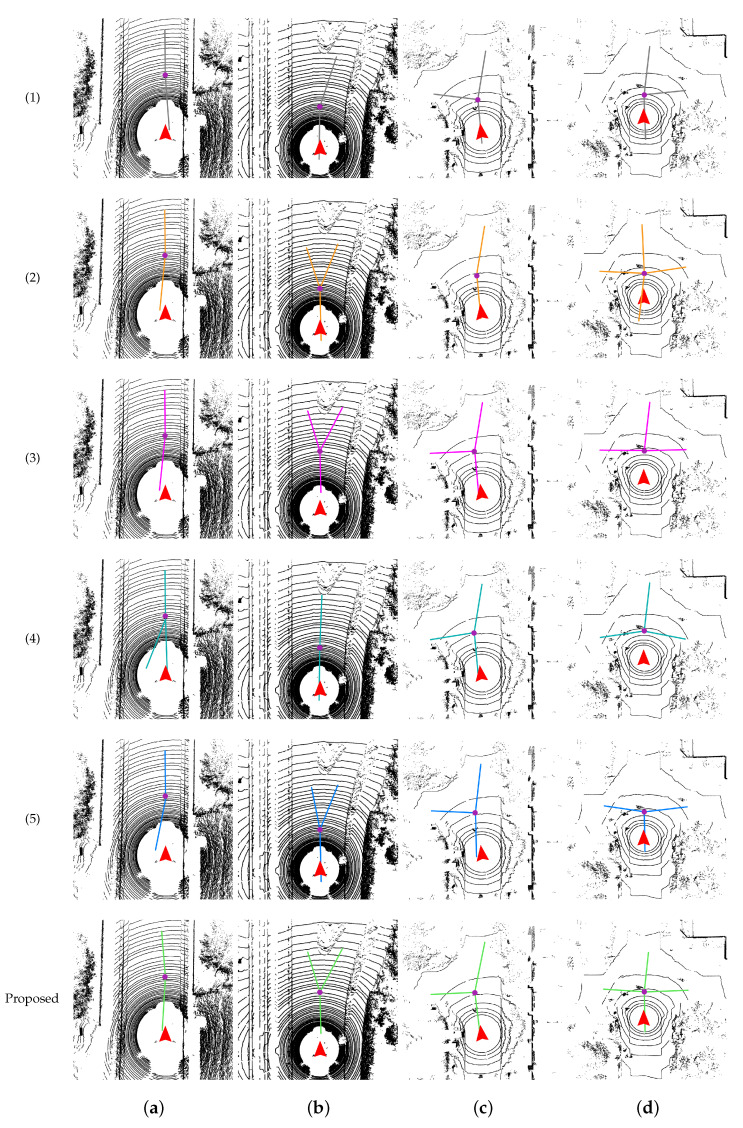
The performance of six approaches on KITTI-raw (Columns (**a**) and (**b**)) and NCP-Intersection (Columns (**c**) and (**d**)) in straight, Y-shaped, T-shaped, and +-shaped intersections, respectively. The first five rows of experimental results correspond to the following five methods. (1): Zhu [8], (2): Chen [7], (3): Zhang [9], (4): Zhang [10], (5): Wang [11].

**Figure 11:**
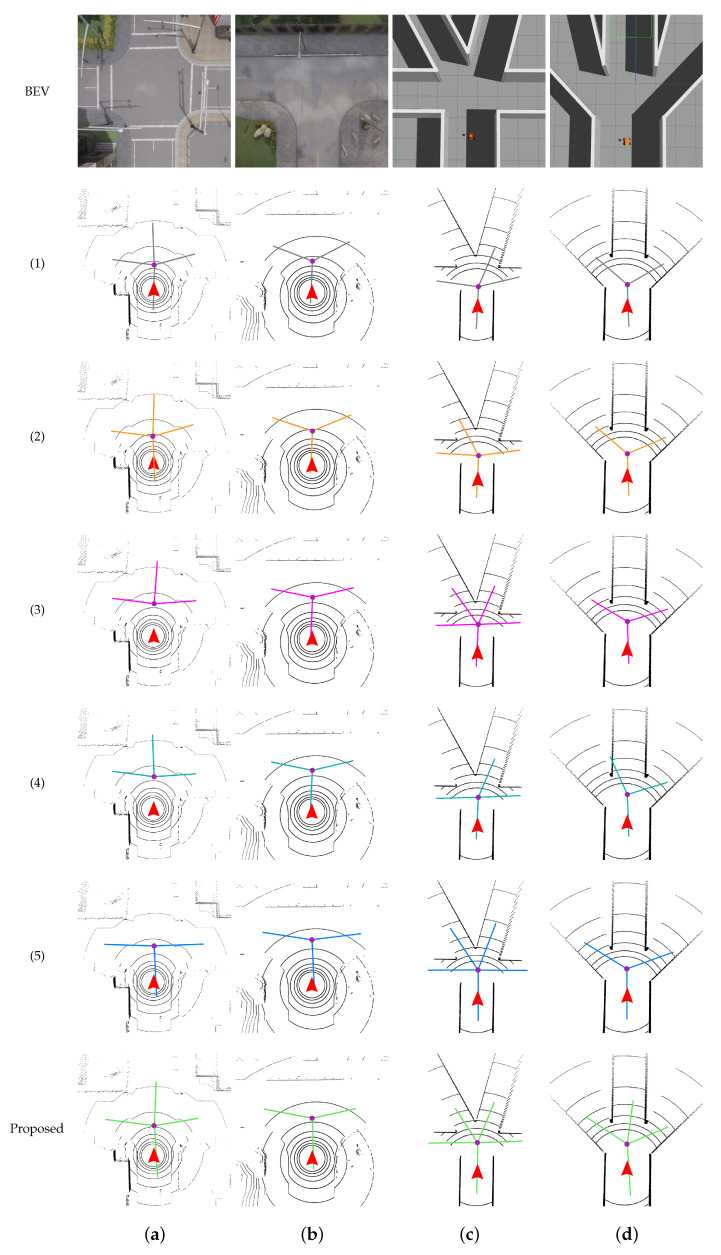
The performance of six algorithms in simulation. Columns (**a**) and (**b**) represent the results obtained in Carla environment, while Columns (**c**) and (**d**) represent the results obtained in Gazebo environment. The experimental results from the second to sixth rows correspond to the following five methods, respectively. (1): Zhu [8], (2): Chen [7], (3): Zhang [9], (4): Zhang [10], (5): Wang [11].

In contrast to the previous method, our approach establishes multiple beam models to minimize false detections. Furthermore, we have designed evaluation metrics to self-assess the quality of the detection results. This allows our method to optimize the detection process in real time. The evaluation is performed on three datasets: KITTI, NCP-Intersection, and the simulation, as illustrated in Figure 10 and Figure 11. To showcase the effectiveness of our method, we select eight frames of representative point cloud images from these datasets. In the figures, the purple points denote the intersection center, while the colored lines indicate different bifurcation directions.

In Figure 10, we have selected four frames from the intersection scenarios, namely, straight, Y-shaped, T-shaped, and +-shaped ones. Zhu [8] adopts a fixed distance for detecting intersections using the beam model, which restricts its ability to detect intersections at different locations. Chen [7] utilizes a range finder-based beam model and employs a distance function in relation to the angle of each beam to identify intersections. While this method shows some improvements, its effectiveness is limited in sparse point clouds, which is particularly evident in Column (c) of Figure 10. The other three comparison methods also exhibit shortcomings when confronted with obstacles. In contrast, our proposed method comprehensively accounts for real-world road conditions and maintains robustness across various structures, regardless of the sparsity of the data source.

Figure 11 displays the detection results in both outdoor flat roads and customized interior structures within the simulation environments. It is observed that as the smoothness of the road surface improves, the detection effects become more robust compared to Figure 10. In the absence of obstacles on an open road, the methods of Zhu [8] and Chen [7] perform relatively well in comparison to the other three methods. However, when encountering unconventional road structures, all five comparison methods show instances of false detection. In contrast, our proposed method effectively addresses the impact of environmental transitions on the detection results. Even when there are obstructions present at the intersection, our method can accurately detect and locate the intersections.

When comparing the overall effects of Figure 10 and Figure 11 side by side, we can observe that the algorithms proposed in [8] and [7] perform well in a fixed distance in front of the UGV, but its effectiveness is limited in other scenarios. The algorithms in [10] and [9] tend to prioritize detection results with a higher number of bifurcations, which may introduce biases. The method described in [11] shows good performance in most road conditions, but it still struggles to completely eliminate the influence of obstacles. In contrast, our proposed method demonstrates excellent robustness and accuracy across all the listed road structures. It takes into account the outputs of multiple beam models and achieves precise results across various types of terrain.

In addition, we also set four indicators to evaluate the intersection detection effects, including the running time, averaging detecting distance (Dist), intersection segment rate (ISR), and location failure rate (LFR). The ISR focuses on determining whether the detected intersection bifurcation angle is correct, and the LFR records the probability of location estimation failure. Their definitions are shown in Equation (10).

(10) $$\left\{\begin{array}{cc} \mathrm{ISR}=N_{a}/M & \\ \mathrm{LFR}=1-N_{t}/N_{a} \end{array}\right.$$

*M* is the set of all the sparse point clouds and $N_{a}$ denotes the correct dataset of the detected bifurcation angle results. $N_{t}$ represents the dataset with the correct intersection position. Moreover, commonly used evaluation metrics such as the $F_{1}$ score, Precision (Positive Predictive Value, PPV), and Recall (True Positive Rate, TPR) are also applied. PPV denotes the proportion of correctly detected intersections out of all the detected samples. TPR represents the proportion of correctly detected results out of the labeled intersections. The $F_{1}$ score is defined as the harmonic average of the PPV and TPR. The definitions are shown as follows:

(11) $$\left\{\begin{array}{cc} \mathrm{PPV}=\mathrm{TP}/(\mathrm{TP}+\mathrm{FP}) & \\ \mathrm{TPR}=\mathrm{TP}/(\mathrm{TP}+\mathrm{FN}) & \\ F_{1}=2*\mathrm{PPV}*\mathrm{TPR}/\left(\mathrm{PPV}+\mathrm{TPR}\right) \end{array}\right.$$

where TP is the true positive number and FP is the false positive number (false detection). FN means false negative (missed detection). The detection and location results are shown in Table 2. From the results, we found that while the single beam model in [11] exhibits good real-time performance similar to our method, its detection accuracy is significantly lower compared to our method. The algorithm in [10] is more suitable for the platform with high computing performance and is sensitive to the interference of obstacles. When our method detects an intersection, the UGV is positioned at the farthest distance from the intersection. Despite the point cloud density of KITTI being approximately four times that of NCP-Intersection, the computational time of the proposed algorithm is only 1.3 times when using a grid map. In comparison, our method achieves a faster processing time and performs better across various common road shapes.

## 4. Conclusions

This paper presents a novel and efficient lidar-based algorithm for detecting and locating intersections. The proposed algorithm incorporates the augmented viewpoints beam model, which enhances the robustness of intersection detection in real traffic scenarios involving pedestrians and vehicles. Additionally, an evaluating confidence metric is introduced to enable online self-assessment of the detection performance. Due to the limited availability of datasets for intersection detection, we have taken the initiative to collect and annotate a VLP-16 point cloud dataset that is specifically tailored for intersections. The experimental evaluations conducted on three datasets demonstrate the real-time capability of the proposed method on both X86 and ARM CPUs.

As of now, there is a lack of proposed deep learning-based intersection detection methods that solely rely on point cloud data as input. This is primarily due to the variability in point cloud data across different road structures. Nonetheless, deep learning exhibits notable advantages in terms of feature representation ability when compared to traditional methods. Therefore, there is immense potential for enhancing intersection detection methods by leveraging deep learning techniques. Moving forward, our future work aims to further advance intersection perceptual tasks by integrating additional intersection semantic features through deep learning.

## Figures and Tables

**Table 1:** Mean TSR for various scenarios (bold: best).

		$T_{s}=tan47$	$T_{s}=tan48$	$T_{s}=tan49$	$T_{s}=tan50$	$T_{s}=tan51$
$T_{W}=19$	$T_{L}=29$	0.9780	0.9813	0.9844	0.9832	0.9818
	$T_{L}=30$	0.9834	0.9855	0.9895	0.9876	0.9861
	$T_{L}=31$	0.9872	0.9889	0.9931	0.9917	0.9891
	$T_{L}=32$	0.9905	0.9958	0.9987	0.9941	0.9923
$T_{W}=20$	$T_{L}=29$	0.9842	0.9945	0.9963	0.9932	0.9895
	$T_{L}=30$	0.9873	**1.0000**	**1.0000**	**1.0000**	0.9923
	$T_{L}=31$	0.9858	0.9986	**1.0000**	**1.0000**	0.9902
	$T_{L}=32$	0.9841	0.9918	0.9959	0.9940	0.9897
$T_{W}=21$	$T_{L}=29$	0.9813	0.9842	0.9933	0.9897	0.9822
	$T_{L}=30$	0.9841	0.9956	**1.0000**	0.9935	0.9864
	$T_{L}=31$	0.9867	0.9902	0.9945	0.9879	0.9823
	$T_{L}=32$	0.9822	0.9884	0.9902	0.9843	0.9786

**Table 2:** Average of metrics on three datasets (bold: best).

Dataset	Method	Running Time (ms)↓		Dist (m)↑	ISR↑	LFR↓	TPR↑	PPV↑	$F_{1}$↑
		**on x86**	**on ARM**						
KITTI-raw	Zhu [8]	232	349	4.5	0.6146	0.4223	0.6329	0.6602	0.6463
	Chen [7]	298	412	5.0	0.6786	0.4820	0.6687	0.6823	0.6754
	Zhang [9]	382	686	6.5	0.7233	0.4156	0.7045	0.7298	0.7169
	Zhang [10]	739	996	7.5	0.8483	0.4409	0.7341	0.7563	0.7450
	Wang [11]	118	218	6.0	0.5513	0.7180	0.6231	0.6472	0.6349
	Proposed	**112**	**201**	**8.5**	**0.9231**	**0.1205**	**0.8217**	**0.8486**	**0.8349**
NCP- Intersection	Zhu [8]	158	245	4.5	0.5987	0.4102	0.6163	0.6299	0.6230
	Chen [7]	196	281	5.0	0.6525	0.4657	0.6472	0.6701	0.6585
	Zhang [9]	249	423	6.5	0.7019	0.3782	0.6829	0.7133	0.6978
	Zhang [10]	136	218	7.5	0.8087	0.4213	0.7938	0.8212	0.8073
	Wang [11]	**82**	**138**	6.0	0.5870	0.7483	0.6958	0.7119	0.7038
	Proposed	88	158	**8.5**	**0.9180**	**0.1324**	**0.8548**	**0.8836**	**0.8690**
Simulation	Zhu [8]	154	231	4.5	0.6057	0.4068	0.6241	0.6487	0.6362
	Chen [7]	201	287	5.0	0.6621	0.4544	0.6524	0.6813	0.6665
	Zhang [9]	246	417	6.5	0.7134	0.3675	0.6786	0.7012	0.6897
	Zhang [10]	138	223	7.5	0.8147	0.4398	0.8217	0.8459	0.8336
	Wang [11]	**84**	**145**	6.0	0.5919	0.7462	0.7125	0.7289	0.7206
	Proposed	90	153	**8.5**	**0.9147**	**0.1313**	**0.8856**	**0.9037**	**0.8946**

## Data Availability

Not applicable.
